# Simulating Stress–Strain Behavior by Using Individual Chains: Uniaxial Deformation of Amorphous Cis- and Trans-1,4-Polybutadiene

**DOI:** 10.3390/polym15061441

**Published:** 2023-03-14

**Authors:** Suvrajyoti Kar, Julie L. Cuddigan, Michael L. Greenfield

**Affiliations:** Department of Chemical Engineering, University of Rhode Island, Kingston, RI 02881, USA

**Keywords:** uniaxial deformation, Gaussian chain, rotational isomeric state, polybutadiene, stress–strain

## Abstract

This work develops a probability-based numerical method for quantifying mechanical properties of non-Gaussian chains subject to uniaxial deformation, with the intention of being able to incorporate polymer–polymer and polymer–filler interactions. The numerical method arises from a probabilistic approach for evaluating the elastic free energy change of chain end-to-end vectors under deformation. The elastic free energy change, force, and stress computed by applying the numerical method to uniaxial deformation of an ensemble of Gaussian chains were in excellent agreement with analytical solutions that were obtained with a Gaussian chain model. Next, the method was applied to configurations of cis- and trans-1,4-polybutadiene chains of various molecular weights that were generated under unperturbed conditions over a range of temperatures with a Rotational Isomeric State (RIS) approach in previous work (*Polymer* **2015**, *62*, 129–138). Forces and stresses increased with deformation, and further dependences on chain molecular weight and temperature were confirmed. Compression forces normal to the imposed deformation were much larger than tension forces on chains. Smaller molecular weight chains represent the equivalent of a much more tightly cross-linked network, resulting in greater moduli than larger chains. Young’s moduli computed from the coarse-grained numerical model were in good agreement with experimental results.

## 1. Introduction

Polybutadiene is an important commercial polymer with pertinent applications in the automobile industry. It is such an essential ingredient in rubber tires that they utilize much of the polybutadiene manufactured worldwide. Additional product uses include toughened plastics, gaskets, shock absorbers, shoe soles, and golf balls [1,2].

As a tire rolls, stresses on the tread exert strains through both elastic and viscous mechanisms. The latter results in energy dissipation leading to rolling resistance, which arises from the viscoelastic energy lost at low frequencies during flattening and re-rounding of tires. When a vehicle is in motion, the tire tread flattens against the road and deforms elastomers locally, which results in chains undergoing conformational changes [3,4,5] that alter the number of ways that they can be arranged. This change in arrangement of elastomer chains affects their elastic free energy, which is logarithmically related to the probability distribution of chain end-to-end vectors [3]. As rotation continues, chains and particles in a now-unstressed portion of the tire tread relax as random fluctuations restore the original distribution of chain conformations. This cyclic change in free energy requires work, which is dissipated to some extent as heat that leads to rolling resistance. The magnitudes of these mechanical forces depend on equilibrium shape properties of polymer chains [6]. Thus, studying chain conformations and their changes under deformation is of the utmost importance to understanding the viscous mechanisms at play within rubber tires.

This paper is part of an overall project aimed toward studying elastomer chain conformations and their role in computing rubber viscoelastic properties as they arise in tires. In this work, we describe a novel numerical method for simulating mechanical properties of elastomer chains subjected to deformation. The numerical method arises from a probability-based numerical approach for evaluating the elastic free energy change of chain end-to-end vectors under deformation. We employ uniaxial deformation as a first example.

The intent of the methodology is to obtain mechanical properties from conformational statistics in a numerical approach that can incorporate effects of specific polymer–polymer and polymer–filler interactions. Motivational examples from experiments include functionalized end-groups that provide interactions with silica fillers [7,8], which have been demonstrated to improve metrics that correlate with lower rolling resistance [9,10,11]. The basis of the approach developed here on chain conformation probability density enables sampling over many chain configurations that can be computed without necessarily requiring costly bulk phase molecular dynamics simulations. Instead, the details of chain–chain and chain–filler interactions are coarse grained into the conformational statistics of end-to-end vectors of individual chains.

The static, dynamic, and mechanical properties of polymers are typically described via scaling relationships [12] that apply in the limit of long chain lengths. The entanglement molecular weight can be inferred from the plateau modulus for stress relaxation of chains without cross links [6]. For cross-linked networks, a rubbery plateau can involve both entanglements and cross links, depending on their relative concentrations [13]. For entangled chains, experiments demonstrate [14] that melt viscosity and self-diffusion coefficient scale consistently with the longest relaxation time. Chains or chain segments that are shorter, yet still within a scaling regime, follow Rouse model dynamics.

The corresponding entanglement length scales for polybutadiene depend on temperature and to some extent on measurement technique. Literature sources [6,15] with a basis in rheology report entanglement molecular weights of 2347 and 1543 g/mol (43 and 29 repeat units) for polybutadiene comprised of 96% cis or of 40% cis, 50% trans, and 10% vinyl, respectively. In contrast, flexible end “sub molecules” of 3300 g/mol (61 repeat units) were reported by Cohen Abbad and Guillermo [16] within NMR studies of segment relaxation time in polybutadiene with 68% cis, 27% trans, and 5% vinyl units.

In the work here, we invoke deformations of chains that do not incorporate cross links or explicit entanglements along a chain. Thus we consider our simulation approach to be relevant for chains or chain segments up to the entanglement molecular weight. Deformation of longer chains requires additional considerations that are not made here.

An important question is the extent that macroscale deformation invokes changes on the molecular structure of elastomers. Characterization methods for polymer networks have been reviewed recently [17]. Among prior experimental studies, pertinent information for the calculations performed here comes from characterizations that employed NMR, X-ray scattering, and neutron scattering.

NMR measurements can be sensitive to local relaxation of chain segments that are between cross links or entanglements; thus, they can probe their influences on elasticity [18,19]. Solid-state ${}^{13}$C NMR experiments by Kameda and Asakura [20] on natural rubber under uniaxial deformation demonstrated that many chains retained a capability to relax the ${}^{13}$C chemical shift after macroscopic deformation; they were not reoriented on a molecular scale. However, mobility of amorphous chains increased immediately after deformation was imposed and then relaxed toward the mobility of unstretched samples over long times (ca. 1 h). Valić et al. [21] studied short perdeuterated polybutadiene probes in cross-linked polybutadiene networks. ${}^{2}$H NMR peaks broadened with increasing uniaxial deformation, showing increased anisotropy in the local environment that differed for cis-1,4 vs. trans-1,4 repeat units. Individual segments in the short chains sensed a local change upon deformation. Valić [22] studied ${}^{2}$H relaxation in natural rubber networks that were cross linked while the original material was under deformation at a deformation ratio of $\lambda =L/L_{0}=1.5$. The rubber exhibited orientational correlations that distinguished directions parallel vs. perpendicular to the initial deformation, and these differences increased upon further deformation of the cross-linked chains. Multiple quantum NMR enables separating ${}^{1}$H contributions from elastically inactive chains and probing the extent that chain segments deform locally during macroscopic deformation or swelling [23,24,25]. In cross-linked natural rubber, these local changes were shown [24,25] to be smaller than those predicted for affine deformation or phantom models of networks [26]. Instead, they suggested constraints of the tubes imposed by surrounding chains [3,27,28,29]. Similar results that favor the relatively smaller deformations of the tube model, compared to affine deformation, were shown for shear and compression of lightly cross-linked elastomers [30]. Alternate explanations were mentioned in support of heterogeneous distributions of local forces that rely on deforming only a small fraction of elastically active chains [30]. In summary, some NMR methods support local chain orientation changes in response to deformation. Other methods suggest that little chain rearrangement occurs.

Synchrotron X-ray scattering measurements during elastomer deformation distinguish among amorphous, ordered amorphous, and crystalline regions. Toki et al. [31] found that ∼75% of chains in natural rubber remained unoriented at a strain of 600%. Approximately 5% became oriented with the deformation (while remaining amorphous) and another 20% of chains underwent stress-induced crystallization. The oriented amorphous chains and the crystallites were thought to convey the imposed stress. Neuefeind et al. [32] used synchrotron X-rays on cross-linked siloxane systems and deconvoluted isotropic and angular-dependent contributions to the scattering. They found a slight broadening of bond length and bond angle distributions at Q>6 Å${}^{-1}$. For results that depended on scattering angle, distances increased slightly between chains in directions perpendicular to a uniaxial deformation. Very slight preferential chain alignment (2% and less) with the stretching direction was found at a deformation ratio λ=2.

Small angle neutron scattering (SANS) enables computing the average radius of gyration $r_{g}$ of chain conformations [33]. Beltzung et al. [34] used SANS to measure changes in $r_{g}$ parallel and perpendicular to the deformation direction for end-linked siloxane chains. Changes in shape were closer to those of a phantom model and were smaller than predicted by an affine deformation. This smaller expansion was more apparent for chains of higher molecular weight. Fernandez et al. [35] cross linked deuterated polybutadiene into polybutadiene. During swelling with toluene, expansion occurred by a factor that followed the phantom model rather than the larger expansion of affine deformation. Straube et al. [36] measured deuterated isoprene strands cross linked into a polyisoprene network. Changes in the two-dimensional scattering pattern were consistent with a tube model of deformation in which tube diameter scaled with $\lambda^{1/2}$ rather than scaling affinely with deformation.

Measurements of mechanical properties provide data and directly test stress–strain models. Dossin and Graessley [37] synthesized various polybutadiene compositions (40–55% cis, 51–38% trans, 9–7% vinyl) and used radiation to create cross-linked networks. Then, they measured uniaxial deformation and interpreted their results in terms of a phantom network model. Both cross links and entanglements contributed to elastic modulus in each network. Subhani et al. [38] performed uniaxial tension and compression tests on sheet-shaped rubber-like materials and developed a stored energy function to predict such experimental results. Their proposed model predicted tension behavior accurately for either uniaxial tension or compression. Starkova and Aniskevich [39] carried out uniaxial tension tests on silica-filled SBR rubbers in order to compare the dependency of Poisson ratio on the definition of finite strain. When using Hencky strain, $\epsilon_{H}\equiv \ln\lambda$, they found that this rubber system was incompressible up to a deformation ratio λ=4.5 and thus corresponded to a Poisson ratio of 0.5. Beyond a deformation of 4.5, the Poisson ratio decreased toward 0.4. Poisson ratio appeared strain-dependent when it was computed from engineering strain. Tonelli and Andrady created PDMS networks in which the molecular weight between cross links was tightly controlled through the synthesis mechanism [5,40]. The modulus of a network with nine repeat units between cross links was ca. 20 times higher than a typical PDMS modulus in which ca. 200 repeat units arise between cross links.

Some studies combine multiple experimental methods. Chen et al. [41] focused on strain-induced crystallization during uniaxial deformation of cross-linked polybutadiene rubber. Measurements at 25 °C of extensional rheometry and synchrotron WAXD indicated that fracture at deformation ratio λ=4.1 was accompanied by the presence of an oriented amorphous phase. Strain-induced crystallization occurred during uniaxial deformation at temperatures of 0 °C to −30 °C and was accompanied by much larger elongations before break. Engineering stress depended linearly on Hencky stress up to $\epsilon_{H}\approx 0.8$ (λ≈2.2). Stresses measured with the extensional rheometer followed the same trends as stress measured with a universal testing machine yet was ∼1.75 times larger.

Many insights about relationships between elastomer chain conformation and mechanical properties have been obtained through molecular dynamics simulations of generic chain representations. Svaneborg et al. [42] simulated the deformation of cross-linked coarse-grained chains of various strand lengths. The extent of chain deformation with strain varied along the strand contour, reaching affine deformation at the end-to-end distance between cross links. The magnitude of stress response was consistent with a double tube model of cross link and entanglement contributions. Simulations of extension by Hsu and Kremer [43], sampled in large systems of entangled chains, exhibited heterogeneous force distributions along an average chain contour. Their results suggested that entanglements could control how stress is maintained across chains.

Many simulations target polybutadiene specifically. Tsolou et al. [44] simulated cis-1,4-polybutadiene for times up to 600 ns over temperatures of 298 to 430 K. Density and its temperature dependence were consistent with prior simulations and with experimental data [45]. Transitions from Rouse-like motions to reptation were found to occur around a chain length of 200 carbon atoms (50 repeat units). They also summarized prior MD simulations of polybutadiene and additional experimental literature relevant for polybutadiene viscoelasticity. In one of those prior efforts, Gee and Boyd [46] simulated cis- and trans-polybutadiene using a united atom model. They found that torsional rotation rates were similar in isolated chains and in the bulk. Thermal expansion coefficient was similar to experimental data [45], while specific volumes were smaller. In more recent efforts, Behbahani et al. [47] simulated polybutadiene–silica nanocomposites by atomistic MD. Characteristic ratio retained nearly the same value as in the bulk. Adsorption slowed chain dynamics time scales. Changes in diffusion scaling from Rouse-like to reptation-like were still observed, and the transition continued to occur near ∼50 repeat units.

Recent works target parameterizing coarse-grained models to represent atomistic MD results. Pavlov and Khalatur [48] used dissipative particle dynamics (DPD) to simulate cross-linked polybutadiene without and with silica nanoparticles. Their coarse-grained model network corresponded to 40 to 50 units per strand. Systems with silica showed a higher modulus compared to unfilled systems. Young’s modulus from simulation was similar to experimental values when its strain rate dependence was extrapolated to the (much lower) experimental range. Kempfer et al. [49] parameterized a DPD model for polybutadiene by matching an atomistic MD trajectory, which led to temperature-dependent parameters. Microsecond simulations on long polybutadiene chains reached a plateau modulus at relaxation times of ∼0.1 μs; it was comparable in magnitude to experimental data. Behbahani et al. [50] linked united atom, coarse-grained, and slip-link simulations to span wide ranges of molecular weight and simulation time for cis-1,4-polybutadiene. Predictions of stress relaxation and storage and loss moduli were similar to experimental data. Physically meaning time shifts among methods were consistent across multiple dynamical properties. End-to-end vector rotations were most important for matching the most coarse-grained method to more detailed molecular time scales.

Some models and simulations target conformations of individual chains. In the Rotational Isomeric State (RIS) approach [5,51], torsions about single bonds are treated as existing in one or more discrete rotational states; each state is chosen to coincide with a conformationally important region of low potential energy. Mark [52,53] used the RIS approach to study configurational statistics of single cis- and trans-1,4-polybutadiene chains under theta conditions. Others who used the RIS approach as an effective tool to study single chain properties of polybutadiene include Abe and Flory [54] and Kajiwara and Burchard [55,56]. Tonelli and Andrady used an RIS model for PDMS to compute two modulus estimates, which bracketed the modulus that they measured [5,40]. Mark et al. [57] reviewed ways to incorporate the RIS model with filler particles. In previous work [58], we studied size and shape properties of amorphous cis- and trans-1,4-polybutadiene systems. We generated single chains under unperturbed conditions, as demonstrated by mean-squared end-to-end distance scaling with number of repeat units (scaling exponent ν=0.5). Then, we analyzed single chain size and shape. Characteristic ratios of both cis- and trans-1,4-polybutadiene chains increased with rising temperature, indicating chain swelling. Probability density distributions of chain size over a range of temperatures demonstrated a previously unreported effect: an increase in average chain size with rising temperature originated from increases in the least likely and most taut conformations rather than from uniform changes across the size distribution. We called this a “*taut conformation effect*”. We also found analogous effects for amorphous polypropylene and polystyrene [59].

The present work focuses on demonstrating a numerical method for computing a stress–strain relationship via changes that occur in chain conformational statistics upon a specified macroscale deformation. As a test case, we employ numerical sets of end-to-end distances that follow a Gaussian distribution. These are used to confirm that this numerical deformation methodology obtains the analytical uniaxial stress–strain relationship that is known for Gaussian chains. We then performed computations to predict the stress–strain relationship of unoriented cis- and trans-1,4-polybutadiene chains subjected to uniaxial deformation by using the resulting changes in their chain conformation statistics. Distributions of end-to-end distances of RIS chains, generated and analyzed previously [58] under unperturbed conditions via the RIS approach, provided the input information. Forces, stresses, and moduli as a function of deformation ratio, molecular weight, and temperature constitute the results.

## 2. Methodology

### 2.1. Coarse Graining Steps

The methods explored here employ two levels of coarse graining. First, the molecular details of polymer chain configuration are subsumed into obtaining a probability density distribution P(r) of end-to-end vectors. Second, each polymer chain that undergoes stress is assumed to be in an environment of similar polymer chains within a region that can be defined via a bulk density. The chains in this environment may constitute a bulk short-chain polymer, chain segments between entanglements, or independent sub-chains within a cross-linked polymer network.

A probability distribution of end-to-end distances serves as an input to the mechanical property calculations. The assumptions that are employed in obtaining this distribution provide an outline of the polymer system that is being considered. Initial calculations here use the analytic distribution for a Gaussian chain. Subsequent calculations employ numerical distributions that were obtained earlier [58] via calculations that used an RIS model. The intent of the approach here is to provide a method that can accommodate changes in P(r) that are induced by specific interactions along a chain, such as distinct groups that promote binding to filler particles.

Literature data described above indicate a range of observed chain-level deformations, which depend on chain length and analysis method. As a starting point, we assume affine deformation, i.e., deformation applied macroscopically is transferred uniformly and microscopically to every chain within the ensemble [13,60]. Employing an approximation applied previously by others [6], we attribute an equivalent occupied volume per chain

(1) $$V=L_{x}L_{y}L_{z}=\frac{M}{\rho }\left(\frac{1}{N_{A}}\right)$$

that incorporates temperature-dependent density ρ (g/cm^3^) and molecular weight *M* (g/mol) of an elastically active segment. A conversion factor (10^7^ nm/cm^3^) provides molecular-scale units. Densities of cis- and trans-1,4-polybutadiene at different temperatures are available from prior experimental and simulation results [44,45,46], which are consistent with measurements [61] of density over extensive temperature and pressure ranges for 4 polybutadienes that differed in cis/trans/vinyl ratio. To simplify the approach initially, we assume monodisperse chains and homogeneous chain orientations, which provide $L_{x}=L_{y}=L_{z}=L=V^{1/3}$. We note that this volume is smaller than the volume swept out by a chain of this molecular weight with radius of gyration $r_{g}$.

Uniaxial deformation was applied to each chain in an ensemble via extension along the *x*-direction, which we express by the deformation ratio $\lambda_{x}=L_{x}/L_{x,0}\equiv \lambda$. This deformation induces a corresponding deformation along the *y* and *z* directions. Since polybutadiene is an elastomer, we utilized a Poisson ratio (ν) of 0.5 [4,39], which corresponds to neglecting any volume change with strain. The accompanying deformations applied along the *y* and *z* directions are compressions such that $\lambda_{y}=\lambda_{z}=1/\sqrt{\lambda_{x}}$, thereby maintaining $\lambda_{x}\lambda_{y}\lambda_{z}=1$.

These deformations change the cross-sectional areas of the nominal box $\left(L_{x},L_{y},L_{z}\right)$ that is invoked as encompassing each chain. The area with its normal vector aligned with the tension direction varies as $L^{2}/\lambda$; the areas aligned with compression vary as $L^{2}\lambda^{1/2}$. Stresses are expected to occur in the same direction as each deformation, as in a tensile test.

### 2.2. Elastic Free Energy

The approach here to quantify mechanical properties arises from how the elastic free energy of an elastomer changes under deformation. Elastic free energy $A_{el}$ of a single chain of end-to-end vector r is related to the probability density distribution P(r) of its end-to-end vector r [3,51,57]

(2) $$A_{el}=c\left(T\right)-k_{b}T\ln P\left(r\right)$$

where c(T) is only a function of temperature *T* and $k_{b}$ is the Boltzmann constant. An isothermal change in elastic free energy of a single chain from an undeformed state $r_{0}$ to a deformed state r is

(3) $$\Delta A_{el}=A_{el}-A_{el,0}=-k_{b}T\left[\ln P\left(r\right)-\ln P\left(r_{0}\right)\right]$$

For an ensemble containing *N* chains, the average change in elastic free energy per chain is obtained by integrating over all chains. This was achieved by multiplying Equation (3) by the probability density distribution of r and integrating over all possible chain end-to-end vectors,

(4) $$\frac{\langle \Delta A_{el}\rangle }{k_{b}T}=-\int P\left(r\right)\;\ln P\left(r\right)\;dr^{3}+\int P\left(r_{0}\right)\;\ln P\left(r_{0}\right)\;dr_{0}^{3}$$

If a chain start is considered to be the origin, then these correspond to integrals over possible chain end positions. To account for how the elastic free energy change is targeting a chain segment that arises between cross links or between entanglements, it is necessary to include a “front factor” [13] $r^{2}/{\langle r^{2}\rangle }_{0}$ prior to integration, i.e., the ratio of mean-squared end-to-end distance in the cross-linked or entangled network relative to that of unencumbered chains of the same length.

Our objective is to reach a simulation approach that can accommodate cases in which P(r) cannot be found analytically. The presence of specific chain–filler interactions is an example. Numerical integration of Equation (4) is then an option. To reach such a simulation framework, we describe end-group positions of chains through “voxels”, where each voxel represents a finite cube in three-dimensional space that is a possible endpoint for an end-to-end vector. The three-dimensional integral over positions r is approximated by a sum over a countable number of voxels. Assigning the positions of chain ends into voxels corresponds to a numeric approach toward evaluating the free energy.

Such a numerical integration of Equation (4), including the front factor, leads to

(5) $$\frac{\langle \Delta A_{el}\rangle }{k_{b}T}=-\sum_{\mathrm{voxels}}P\left(r\right)\;\ln P\left(r\right)\;\frac{r^{2}}{{\langle r^{2}\rangle }_{0}}\;{\left(\Delta r\right)}^{3}+\sum_{\mathrm{voxels}}P\left(r_{0}\right)\;\ln P\left(r_{0}\right)\;\frac{r_{0}^{2}}{{\langle r^{2}\rangle }_{0}}\;{\left(\Delta r_{0}\right)}^{3}$$

where (${\Delta r)}^{3}={\left(\Delta r_{0}\right)}^{3}$ is the volume of a voxel, $r_{0}^{2}$ and $r^{2}$ represent undeformed and deformed chain end-to-end distances, respectively, and ${\langle r^{2}\rangle }_{0}$ is the average of the mean squared end-to-end distance of the undeformed chains.

To be a simulation, the probability density $P(r_{0})$ of undeformed chains is available in numeric form. Here, simulation involves creating individual chains by using intra- and inter-chain energy functions along with any other specific interactions of interest. This creates a set of *N* independent realizations in the ensemble of single chains, each with their own $r_{0}$ vector.

After deformation, the probability densities P(r) were re-computed for ensembles of chain end-to-end vectors by using voxel location along the x,y, *z* coordinate directions

(6) $$P\left(r\right)dr=\frac{\mathrm{number}\;\mathrm{of}\;\mathrm{chains}\;\mathrm{in}\;\mathrm{voxel}(x,y,z)\mathrm{at}\;r}{\mathrm{volume}\;\mathrm{of}\;\mathrm{voxel}(x,y,z)*N}dr$$

and simulation entails altering an end-to-end vector $r_{0}$ so it represents a new, deformed direction r. Initial test cases used chains with (a) Gaussian end-to-end vectors and (b) chains generated with an RIS approach.

### 2.3. Force Derivatives of Free Energy

Deformation applied to elastomers induces strain, which leads to changes in elastic free energy. Conversely, an induced strain leads to quantifiable forces and stresses. The combination of force and distance (or stress and strain), applied reversibly, corresponds to a thermodynamic work term. Thus force per chain can be related directly to the change in elastic free energy with system dimension as [13]

(7) $$f_{k}=\left(\frac{\partial \lambda_{k}}{\partial L}\right){\left(\frac{\partial \langle \Delta A_{el}\rangle }{\partial \lambda_{k}}\right)}_{T,V}$$

where deformation ratio $\lambda_{k}=r_{k}/r_{k,0}$ [4] applied in the *k*th direction leads to force $f_{k}$ in the *k*th direction. $r_{k}$ and $r_{k,0}$ are components of deformed and undeformed chain end-to-end vectors, respectively. $L_{k}$ refers to a characteristic size of a chain, as described above.

The tensile stress acting on each elastically active chain in the system due to deformation is obtained by dividing the force by the cross-sectional area ($A_{m}$) whose normal vector is aligned with the applied force,

(8) $$\sigma_{mk}=\left(\frac{1}{LA_{m}}\right){\left(\frac{\partial \langle \Delta A_{el}\rangle }{\partial \lambda_{k}}\right)}_{T,V}$$

Choosing actual or initial cross-sectional area leads to Equation (8) providing either a true stress or an engineering stress [13].

We focus in this work on true stress. The true cross-sectional area (in *y*-*z*) decreases with uniaxial extension in *x* and is represented by $L^{2}/\lambda_{x}$. Thus, Equation (8) can be written in the extension direction as

(9) $$\sigma_{xx}=\left(\frac{\lambda_{x}}{V}\right){\left(\frac{\partial \langle \Delta A_{el}\rangle }{\partial \lambda_{x}}\right)}_{T,V}$$

The equivalent volume of a single chain (*V*) was defined above. For transverse (compression) directions, the true cross-sectional area increases and can be represented by $L^{2}{\lambda_{x}}^{1/2}$, leading to

(10) $$\sigma_{yy}=\left(\frac{1}{V{\lambda_{x}}^{1/2}}\right){\left(\frac{\partial \langle \Delta A_{el}\rangle }{\partial \lambda_{y}}\right)}_{T,V}$$

with an analogous equation in the *z* direction.

Tension force in the *x* direction can be obtained numerically by substituting the numeric estimate of the elastic free energy per chain, Equation (5), into the force derivative, Equation (7), as

(11) $$\frac{f_{x}}{k_{b}T}=\frac{1}{L}{\left(\frac{\partial \left(-\sum_{\mathrm{voxels}}P\left(r\right)\;\ln P\left(r\right)\;\frac{r^{2}}{{\langle r^{2}\rangle }_{0}}\;{\left(\Delta r\right)}^{3}+\sum_{\mathrm{voxels}}P\left(r_{0}\right)\;\ln P\left(r_{0}\right)\;\frac{r_{0}^{2}}{{\langle r^{2}\rangle }_{0}}\;{\left(\Delta r_{0}\right)}^{3}\right)}{\partial \lambda }\right)}_{T,V}$$

where the difference in the numerator corresponds to simultaneous deformations λ that involve changes in all three directions, i.e., extension along the *x*-direction and compression along the *y*- and *z*-directions.

For compressive forces in transverse directions, the differential change in deformation ratio can be replaced by invoking the chain rule, $\partial \lambda_{y}=\partial \left(1/\sqrt{\lambda }\right)=-\left(1/2\lambda^{3/2}\right)\partial \lambda$, leading to

(12) $$\frac{f_{y,z}}{k_{b}T}=-\frac{2\lambda^{3/2}}{L}{\left(\frac{\partial \left(-\sum_{\mathrm{voxels}}P\left(r\right)\;\ln P\left(r\right)\;\frac{r^{2}}{{\langle r^{2}\rangle }_{0}}\;{\left(\Delta r\right)}^{3}+\sum_{\mathrm{voxels}}P\left(r_{0}\right)\;\ln P\left(r_{0}\right)\;\frac{r_{0}^{2}}{{\langle r^{2}\rangle }_{0}}\;{\left(\Delta r_{0}\right)}^{3}\right)}{\partial \lambda }\right)}_{T,V}$$

where $f_{y,z}$ are compression forces per chain in the *y* and *z* directions, respectively.

Numerical equations for tensile and compressive true stresses are obtained by dividing tension and compression forces per chain with their respective instantaneous cross-sectional areas, which are $L_{y}L_{z}=L^{2}/\lambda$ for tension and $L_{x}L_{y}=L_{x}L_{z}=L^{2}\sqrt{\lambda }$ for compression, leading to

(13) $$\frac{\sigma_{xx}}{k_{b}T}=\frac{\lambda }{V}{\left(\frac{\partial \left(-\sum_{\mathrm{voxels}}P\left(r\right)\;\ln P\left(r\right)\;\frac{r^{2}}{{\langle r^{2}\rangle }_{0}}\;{\left(\Delta r\right)}^{3}+\sum_{\mathrm{voxels}}P\left(r_{0}\right)\;\ln P\left(r_{0}\right)\;\frac{r_{0}^{2}}{{\langle r^{2}\rangle }_{0}}\;{\left(\Delta r_{0}\right)}^{3}\right)}{\partial \lambda }\right)}_{T,V}$$

(14) $$\frac{\sigma_{yy,zz}}{k_{b}T}=-\frac{2\lambda }{V}{\left(\frac{\partial \left(-\sum_{\mathrm{voxels}}P\left(r\right)\;\ln P\left(r\right)\;\frac{r^{2}}{{\langle r^{2}\rangle }_{0}}\;{\left(\Delta r\right)}^{3}+\sum_{\mathrm{voxels}}P\left(r_{0}\right)\;\ln P\left(r_{0}\right)\;\frac{r_{0}^{2}}{{\langle r^{2}\rangle }_{0}}\;{\left(\Delta r_{0}\right)}^{3}\right)}{\partial \lambda }\right)}_{T,V}$$

The net result of these derivations are equations that can be implemented numerically for strains that deform an unperturbed polymer with distribution $P(r_{0})$ into a deformed system with probability density distribution P(r).

### 2.4. Orientation of Undeformed and Deformed Chains

Polymer chains within a bulk system are oriented in a manner that reflects their processing and sample preparation. In this work, we develop the method first by employing a homogeneous system of unoriented chains in the absence of entanglements.

To evaluate Equations (11)–(14), the orientations of individual chain end-to-end vectors within an ensemble have to be distributed over a three-dimensional space. Starting from a set of end-to-end distances [58], we oriented chain conformations randomly over the (+x,+y,+z) octant of a sphere prior to imposing deformation. Distributing the chains over a single octant of a sphere assumes that the same distribution occurs along the remaining sections of the sphere, and it provides better statistics for a given ensemble population and computation time.

Chain orientation was accomplished by considering each end-to-end distance as a randomly distributed unperturbed end-to-end vector in spherical coordinates,

(15) $$\left(r_{x,0}\;,r_{y,0}\;,r_{z,0}\right)=r_{0}\left(\sin\theta \cos\phi \;,\sin\theta \sin\phi \;,\cos\theta \right)$$

Each elevation or latitudinal angle θ∈[0,π/2] was chosen uniformly from a sinusoidal distribution between [0,1]. Each longitudinal angle ϕ was chosen uniformly within [0,π/2).

Chain deformation was implemented by applying a deformation ratio, representative of either extension ($\lambda_{x}\equiv \lambda$) or compression ($\lambda_{y,z}=1/\sqrt{\lambda }$), to each vector component in its respective *x*, *y*, and *z* direction. Thus, each end-to-end vector of a chain after deformation was represented as

(16) $$\left(r_{x}\;,r_{y}\;,r_{z}\right)=r_{0}\left(\lambda \sin\theta \cos\phi \;,\frac{1}{\sqrt{\lambda }}\sin\theta \sin\phi \;,\frac{1}{\sqrt{\lambda }}\cos\theta \right)$$

Probability density distributions for the deformed end-to-end vectors were obtained by averaging over the number of chains ending in the region around each r. For the homogeneous case, the results from this one octant should be equivalent to results for all other octants. The outcome is the probability density distribution for uniaxial deformation to a deformation ratio λ.

### 2.5. Numerical Implementation

Deformations were performed in steps of Δλ=0.25 from λ=1 to 3.5 to obtain discrete changes in average elastic free energy per chain $\langle \Delta A_{el}\rangle$ via Equation (5). The numerical derivatives of Equations (11)–(14) were obtained by central difference, i.e., changes in the numerator and denominator between (λ+Δλ) and (λ−Δλ).

### 2.6. Gaussian Test Case

In order to verify the accuracy of the proposed numerical method, we applied it to Gaussian chains and compared the results with analytical solutions. The probability density distribution for the end-to-end vector $r_{0}$ of a Gaussian chain of mixed single and double bonds in the absence of imposed stress is written analytically as [51]

(17) $$P\left(r_{0}\right)dr_{0}={\left(\frac{3}{2\pi C_{n}\left(n_{s}l_{s}^{2}+n_{d}l_{d}^{2}\right)}\right)}^{3/2}\exp\left(\frac{-3r_{0}^{2}}{2C_{n}\left(n_{s}l_{s}^{2}+n_{d}l_{d}^{2}\right)}\right)4\pi {r_{0}}^{2}dr_{0}$$

where $r_{0}$ is the end-to-end distance of a chain, $C_{n}$ is the characteristic ratio [51], $n_{i}$ is the number of backbone bonds of each type along a polymer chain, $l_{i}$ is the bond length, and subscripts *s* and *d* indicate single and double bonds where $l_{s}$ = 1.53 Å and $l_{d}$ = 1.10 Å for polybutadiene [52].

To replicate the use of a simulation, an ensemble of $N=10^{5}$ end-to-end distances was created that satisfies this distribution. Specifically, independent individual Gaussian chains were represented by repeatedly determining the distance *r* that satisfies

(18) $$\xi ={\left(\frac{3}{2\pi C_{n}\left(n_{s}l_{s}^{2}+n_{d}l_{d}^{2}\right)}\right)}^{3/2}\int_{0}^{r}\exp\left(\frac{-3r_{0}^{2}}{2C_{n}\left(n_{s}l_{s}^{2}+n_{d}l_{d}^{2}\right)}\right)4\pi r_{0}^{2}dr_{0}$$

where ξ was chosen uniformly within [0,1). A linear congruential random number generator was used to generate the sequence of $10^{5}$ values of ξ. A different seed was used for each molecular weight, temperature, and chain type to ensure independent statistics.

The change in elastic free energy for an ensemble of Gaussian chains is obtained by substituting Equation (17) in Equation (3) and averaging over all chains, leading to [3]

(19) $$\frac{\langle \Delta A_{el}\rangle }{k_{b}T}=\frac{3}{2}\left(\frac{\langle r^{2}\rangle }{{\langle r^{2}\rangle }_{0}}-1\right)$$

The mean squared end-to-end distances can be represented in terms of end-to-end vectors as

(20) $${\langle r^{2}\rangle }_{0}={\langle r_{x}^{2}\rangle }_{0}+{\langle r_{y}^{2}\rangle }_{0}+{\langle r_{z}^{2}\rangle }_{0}$$

(21) $$\langle r^{2}\rangle =\langle r_{x}^{2}\rangle +\langle r_{y}^{2}\rangle +\langle r_{z}^{2}\rangle$$

Assuming the initial elastomer system to be isotropic in a state of rest [3] equates the contributions in each direction,

(22) $${\langle r_{x}^{2}\rangle }_{0}={\langle r_{y}^{2}\rangle }_{0}={\langle r_{z}^{2}\rangle }_{0}={\langle r^{2}\rangle }_{0}/3$$

The mean squared end-to-end distance can be written in terms of deformation ratio as

(23) $$\langle r_{i}^{2}\rangle ={\langle r_{i}^{2}\rangle }_{0}\lambda_{i}^{2}$$

where *i* = x,y,z. The free energy change for Gaussian chains can be written in terms of deformation ratios [3] by substituting Equations (20)–(23) in Equation (19)

(24) $$\frac{\langle \Delta A_{el}\rangle }{k_{b}T}=\frac{1}{2}\left(\lambda_{x}^{2}+\lambda_{y}^{2}+\lambda_{z}^{2}-3\right)$$

Under uniaxial deformation λ in the *x* direction, with compression $1/\sqrt{\lambda }$ in the *y*- and *z*-direction in accordance with a Poisson ratio of 0.5, Equation (24) can be written as [4]

(25) $$\frac{\langle \Delta A_{el}\rangle }{k_{b}T}=\frac{1}{2}\left(\lambda^{2}+\frac{2}{\lambda }-3\right)$$

providing an analytic equation for the free energy change of a deformed Gaussian chain. This dependence on λ can be compared with results obtained from using Equation (5), computed using the numerical distribution P(r) tabulated for Gaussian chains, to determine the accuracy of the numerical method for elastic free energy change computations. This dependence on λ relies on applying affine deformation to each chain.

Analytical tension ($f_{x}$) and compression forces ($f_{y,z}$) for an ensemble of Gaussian chains are obtained by applying the force derivative in Equation (7) to the analytic Gaussian expression for free energy, Equation (24), which leads to [13]

(26) $$\frac{f_{x}}{k_{b}T}=\frac{1}{L}\left(\lambda -\frac{1}{\lambda^{2}}\right)$$

(27) $$\frac{f_{y,z}}{k_{b}T}=\frac{2}{L}\left(\frac{1}{\sqrt{\lambda }}-\lambda^{5/2}\right)$$

These may be compared with Equations (11) and (12), respectively, computed using the Gaussian P(r), to verify the numerical method accuracy for force computations.

Analytic tensile ($\sigma_{xx}$) and compressive true stresses ($\sigma_{yy,zz}$) were obtained for the Gaussian chain model by dividing the tension and compression forces by their respective instantaneous cross-sectional areas, leading to

(28) $$\frac{\sigma_{xx}}{k_{b}T}=\frac{1}{V}\left(\lambda^{2}-\frac{1}{\lambda }\right)$$

(29) $$\frac{\sigma_{yy,zz}}{k_{b}T}=\frac{2}{V}\left(\frac{1}{\lambda }-\lambda^{2}\right)$$

These may be compared with Equations (13) and (14) to verify numerical method accuracy for stresses. Substitution for volume by using Equation (1) provides the classic result [13]

(30) $$\frac{\sigma_{xx}}{N_{A}k_{b}T}=\frac{\sigma_{xx}}{RT}=\frac{\rho }{M}\left(\lambda^{2}-\frac{1}{\lambda }\right)$$

An analogous substitution in Equation (13) enables comparisons with experimental stress and modulus measurements.

### 2.7. RIS Chains

Unperturbed RIS chain end-to-end distances ($r_{0}$) from previous work [58] were used as inputs for computing end-to-end vectors of chains before and after deformation. That work provided distributions of amorphous, single RIS chains of cis- and trans-1,4-polybutadiene for different molecular weights (*n* = 15, 25, 50, 75, 100, and 120 repeat units) at a temperature of 343 K as well as at different temperatures (*T* = 275, 300, 323, 343, 375, and 400 K) for a single size of 50 repeat units. Ensembles of Gaussian chains were generated under the same conditions as the RIS chains. Each ensemble consisted of 10${}^{5}$ independent single chains. We found that this size was large enough to provide typical results of sufficient accuracy.

### 2.8. Finite Strain

Engineering strain *e* relates the amount of deformation to the initial size of a sample in that direction. It also relates directly to deformation ratio λ; together, these relations are

(31) $$e=\Delta L/L_{0}=\lambda -1$$

When finite deformation of a three-dimensional body is described in a fully tensorial representation, i.e., changes in its (x,y,z) dimensions with respect to the (x,y,z) directions, the resulting 3×3 strain tensor differs from simpler equations of engineering strain that apply only in the limit of small strain [62].

There are multiple definitions of finite strain that represent deformation more accurately. In the results presented below, we calculate finite strain as [62]

(32) $$\epsilon =\frac{1}{2}\left(\lambda^{2}-1\right)=\frac{1}{2}\epsilon_{e}^{2}+\epsilon_{e}$$

We also express results for Gaussian chains in terms of Hencky strain, calculated as [39] $\epsilon_{H}=\ln\lambda$. Like other definitions, these equate finite strain and engineering strain in the limit λ→1. Slopes of the initial linear regime of stress–strain plots, which employ true stress and finite strain, were used to compute Young’s modulus (*E*) [4,13].

## 3. Results and Discussion

### 3.1. Gaussian Chains

Gaussian chains provide a useful test case because the probability density function for their end-to-end distance can be written analytically. Their elastic energy and stress–strain relationships are also analytic as a consequence, as shown in textbooks [4,13]. Thus, a significant step towards testing the accuracy of the numerical method was comparing numerical results with those analytical results.

Implementing this comparison requires a set of Gaussian chains as an input. For each chain architecture, molecular weight, and temperature, an ensemble of $10^{5}$ independent Gaussian chain end-to-end distances was generated using Equation (18). The probability density distribution of their end-to-end distribution was re-computed for the $10^{5}$ chains.

**Figure 1:**
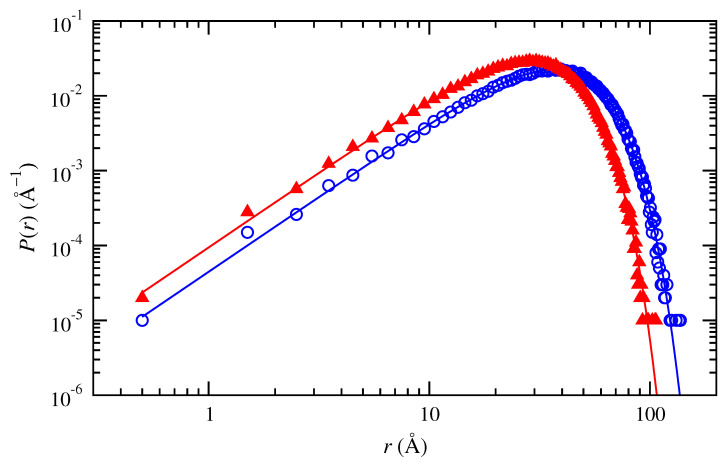
Probability density distributions of end-to-end distance obtained numerically for Gaussian chains of (∘, blue) trans-1,4-polybutadiene and (▴, red) cis-1,4-polybutadiene at 343 K with 50 repeat units. Lines indicate the analytic Gaussian probability density distribution function.

Figure 1 shows good agreement between this numerical distribution for the generated Gaussian chains and the analytic Gaussian probability density distribution at two particular conditions (trans- and cis-1,4-polybutadiene, 50 repeat units, 343 K). Small differences arise at the smallest distances *r*. Here, the discrete probability densities P(r)=(1/Δr)(j/N) that can be achieved for consecutive numbers *j* of chains in a bin of width Δr differ by amounts ±(1/Δr)(1/N) that are comparable in magnitude to the analytic probability density. Equal numerical probability densities at the largest distances *r* correspond to consecutive bins that contain only a single one of the *N* chains in the ensemble.

Uniaxial deformation of this ensemble of chains was considered next. To represent an isotropic initial distribution of chain orientations, the end-to-end vector of each undeformed chain was positioned with one end at (0,0,0). Its other end was oriented randomly within the first quadrant such that the complete set of chain orientation angles sampled a sphere uniformly.

These chains were then uniaxially extended along the *x* direction and compressed along the *y* and *z* directions, leading to each deformed end-to-end vector. These correspond to forces that are aligned with the surface normal directions of a hypothetical parallelepiped that surrounds each chain, e.g., stress $\sigma_{xx}$ and strain $\epsilon_{xx}$. Cumulatively, independent deformation of all chains in the ensemble resulted in a change in their probability density distributions and elastic free energy, which allowed for determining tensile forces and stresses via Equations (11) and (13).

**Figure 2:**
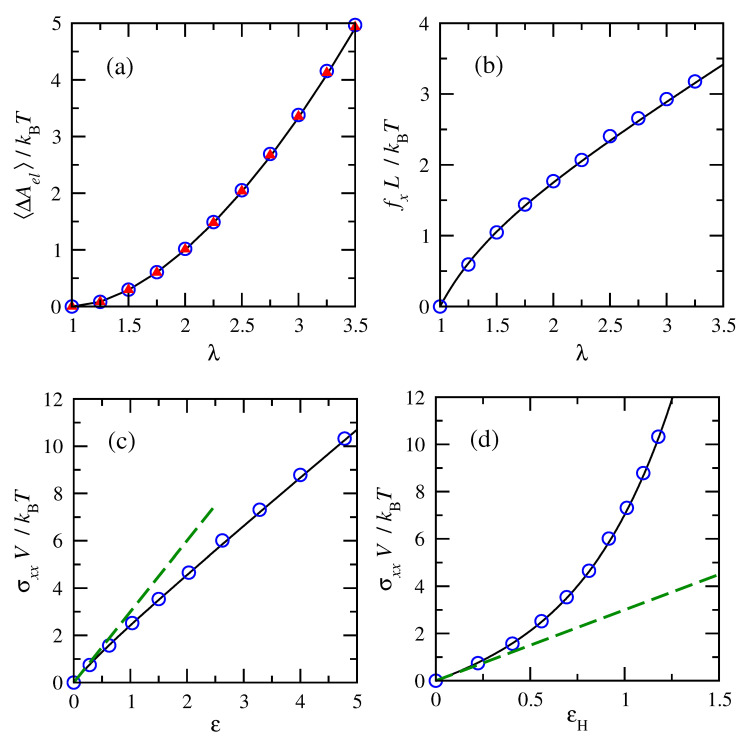
Analytical and numerical comparisons of (**a**) elastic free energy changes, (**b**) force in tension, and (**c**) stress in tension for (∘, blue) trans- and (▴, red) cis-1,4-polybutadiene chains of 50 repeat units at 343 K. The dashed line indicates the initial modulus. Hencky finite strain is employed in (**d**). Overlapping results for cis- are omitted in (**b**–**d**).

Conditions for trans- and cis-1,4-polybutadiene of 50 repeat units at 343 K were selected for displaying a comparison between numerical results and analytical expressions. Figure 2a shows that changes in elastic free energy with deformation were the same (within numerical error) for the trans- and cis- chains, as expected for a Gaussian model. Both agree well with the analytic solution: free energy rises with deformation as $\frac{1}{2}\left(\lambda^{2}+2/\lambda -3\right)$. Thus, only force and stress results for trans-1,4-polybutadiene chains are shown in subsequent numeric tests of the proposed approach. Figure 2b,c shows excellent agreement for the extension force and tensile stress for Gaussian chains as computed analytically or as determined via the new simulation method. Compression forces and stresses in the transverse (y,z) directions (not shown) exhibited similar agreement. Tensile force rises with $\left(\lambda -1/\lambda^{2}\right)$. True tensile stress rises with $\left(\lambda^{2}-1/\lambda \right)$, whose slope initially scales as $\left(\partial \sigma /\partial \epsilon \right)=\left(\partial \sigma /\partial \lambda \right)/\left(\partial \epsilon /\partial \lambda \right)=\left(2+1/\lambda^{3}\right)=3$ in the limit of small deformations (λ→1, dashed line). The initial modulus at zero strain helps to illustrate differences from a linear stress–strain relationship at finite strain. Figure 2d shows that the stress–strain relationship for the Gaussian chain deviates further from linearity when plotted using Hencky strain.

Analogous extents of very good agreement were also observed for Gaussian chains that were representative of cis- and trans-1,4-polybutadiene for all numbers of repeat units and all temperatures that were considered. In summary, a comparative analysis of numerical results and analytical solutions of Gaussian chains indicates that the implemented numerical method determines stress–strain behavior accurately for a well-defined model of the end-to-end distance probability density distribution.

### 3.2. RIS Chains

Next, the new method was applied to RIS chains of cis- and trans-1,4-polybutadiene of different numbers of repeat units at 343 K. Probability densities of the undeformed ensembles of chains were shown in earlier work [58]. We initiated these simulations with isotropic (unoriented) chains and applied affine deformation to each chain.

**Figure 3:**
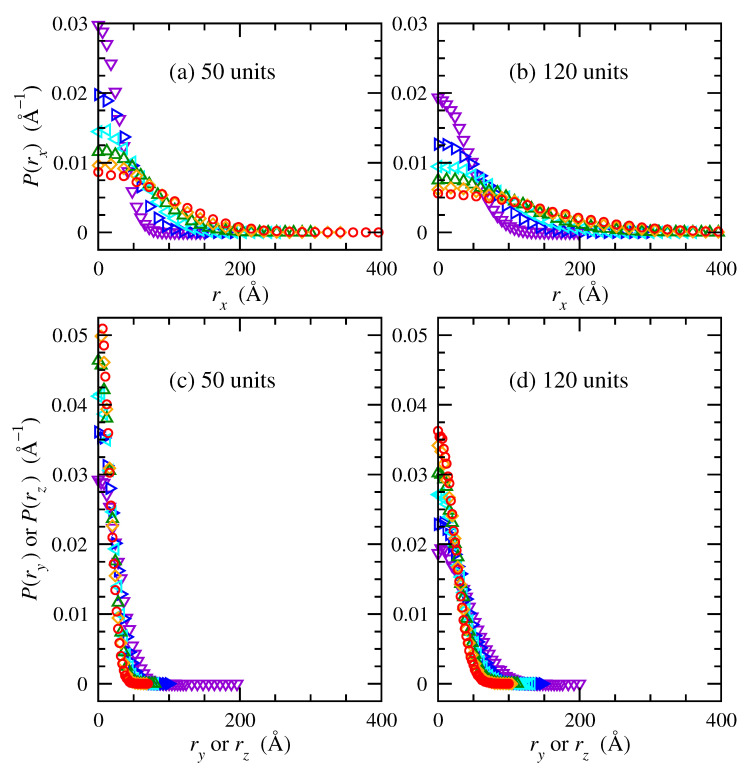
Probability density distributions of end-to-end vectors in the (**a**,**b**) *x* and (**c**,**d**) *y* and *z* directions for trans-1,4-polybutadiene RIS chains of (**a**,**c**) 50, and (**b**,**d**) 120 repeat units at 343 K. Deformation ratios (λ): 1 (▿ violet); 1.5 (▹ blue); 2 (◃ cyan); 2.5 (∆ green); 3 (⋄ orange); 3.5 (∘ red).

Figure 3 shows probability density distributions of end-to-end vector components of trans-1,4-polybutadiene chains of 50 and 120 repeat units under uniaxial tension in directions of extension (panels a, b) and compression (panels c, d). The distributions of $r_{x}$, $r_{y}$, and $r_{z}$ are equivalent under undeformed conditions as a consequence of the isotropic orientation distribution. The $r_{x}$ distribution gradually widens with increasing deformation. This indicates that the highly probable shorter conformations become less probable upon stretching, while longer conformations become increasingly present. Conversely, $r_{y}$ or $r_{z}$ distributions contract in width with increasing deformation; this indicates more probable shorter conformations and less probable longer conformations while they compress in response to extension in the *x* direction.

Chains of 120 repeat units occurred with lower probabilities at any given end-to-end distance, compared to chains of 50 repeat units, because they spanned larger distances. In short, Figure 3 displays explicit changes that occur in contributions to end-to-end distance probability density as a consequence of uniaxial deformation in the *x*-direction applied affinely to each chain.

**Figure 4:**
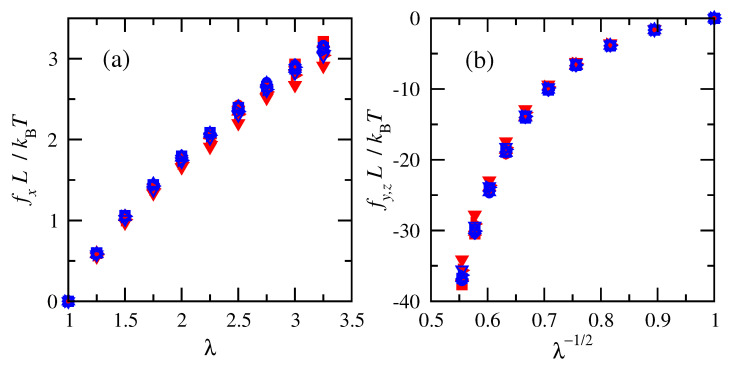
Normalized (**a**) tension and (**b**) compression forces vs. deformation ratio for cis- and trans-1,4-polybutadiene RIS chains of all sizes at 343 K. Filled red symbols for cis and open blue for trans. *n* = 15 (▿), 25 (▹), 50 (▵), 75 (□), 100 (⋄), and 120 (∘).

Numeric changes in the elastic energies as a function of deformation were computed in order to estimate the deformation forces and stresses. Figure 4 shows that normalized tension and compression forces for chains at the same temperature increased with increasing deformation. Much larger forces accompany shrinking the system in the transverse directions than elongating it in the direction of imposed strain, as indicated by the larger magnitudes of compressive forces shown in Figure 4b. This is consistent with the larger forces associated with compression compared to elongation in classic experiments on cross-linked natural rubber [63]. Normalized tensile forces calculated for cis-1,4-polybutadiene are slightly smaller than those calculated for trans-1,4-polybutadiene at high extensions.

Results for different numbers of repeat units (i.e., molecular weights) nearly collapse onto overlapping curves when the physical dimension *L* from the chain density prior to deformation is used to normalize the tension and compression forces. The overlap of the normalized forces indicates that RIS chains of different numbers of repeat units are impacted in the same way by molecular weight and density.

**Figure 5:**
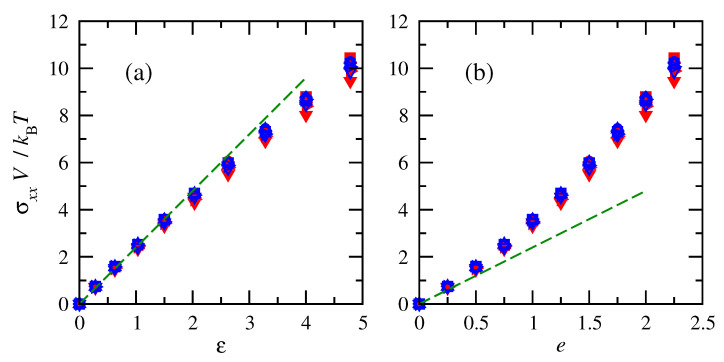
Normalized tensile stresses vs. (**a**) finite strain and (**b**) engineering strain for cis- and trans-1,4-polybutadiene RIS chains of all sizes at 343 K. Symbols follow convention of Figure 4. Dashed lines indicate an initial linear fit with $E=2.4\;k_{B}T/V$.

Similar to the force behavior, tensile stresses acting on the chains increased with increasing deformation (or finite strain), as shown in Figure 5. Normalizations of tensile stresses on the chains demonstrate that across different sizes, stresses are impacted in the same way by molecular weight and density in terms of their combination into chain-occupied volume *V*. Results are similar once again for cis- and trans-1,4-polybutadiene. The stress–strain relationship is closer to linear for finite strain (Equation (32)) compared to engineering strain.

The stress–strain behavior of macroscale elastomers can exhibit features that arise from irreversibilities, strain softening, and hysteresis during multiple stress–strain cycles [38]. The current state of the new simulation method does not enable or account for such changes in chain structure and their effects on end-to-end vector probability density. Thus the mechanical response to deformation is treated reversibly in this work.

**Figure 6:**
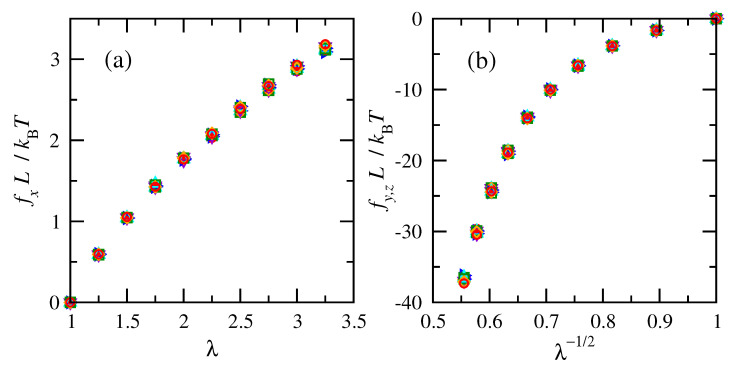
Normalized (**a**) tension and (**b**) compression forces vs. deformation ratio for cis- and trans-1,4-polybutadiene RIS chains of 50 repeat units. Filled symbols for cis and open for trans. *T* = 275 K (▿ violet); 300 K (▹ blue); 323 K (◃ cyan); 343 K (∆ green); 375 K (⋄ orange); 400 K (∘ red).

Figure 6 shows increasing tension and compression forces with increasing uniaxial deformation for chains of the same molecular weight at various temperatures. Each temperature corresponds to a separate distribution of end-to-end distances [58] as inputs to the calculations. Normalization of the forces by the box dimension has the same beneficial effect that was shown in Figure 4. The role of temperature as another normalization term is also clear. While the only difference across chains of the same molecular weight is the temperature, its effects on tension and compression forces is confirmed to be accounted for via this scaling.

**Figure 7:**
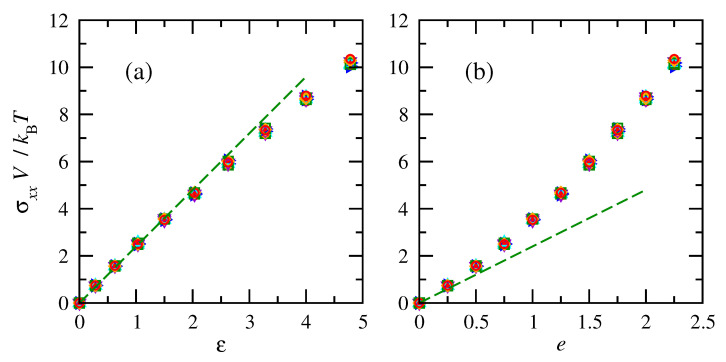
Normalized tensile stresses vs. (**a**) finite and (**b**) engineering strain for cis- and trans-1,4-polybutadiene RIS chains of 50 repeat units. Symbols and colors follow convention of Figure 6. Dashed lines indicate an initial linear fit with $E=2.4\;k_{B}T/V$.

The tensile stresses increased with deformation (or finite strain), as shown in Figure 7. The normalized tensile stresses on chains of the same number of repeat units were consistent across all temperatures. Similar to force, the overlap emphasizes that non-normalized tensile stresses are impacted by temperature. Once again, the stress–strain relationship is closer to linear for finite strain compared to engineering strain. While the simulation results deviate slightly from a linear relationship between true stress and finite strain, they do not indicate a maximum in engineering stress or a yield point. The simulations also cannot detect a macroscopic strain-at-break point.

Two observations can be made in regard to the underlying distribution of chain shapes. Small differences in end-to-end distance probability density distribution with respect to temperature [58], which lead to the temperature dependence of characteristic ratio, do not seem to affect deformation forces or stresses when all chain end-to-end vectors are deformed affinely. Moreover, similar results were found for cis- and trans-1,4-polybutadiene, despite there being some difference in their underlying end-to-end distance distributions, which is manifested in them having different characteristic ratios.

**Figure 8:**
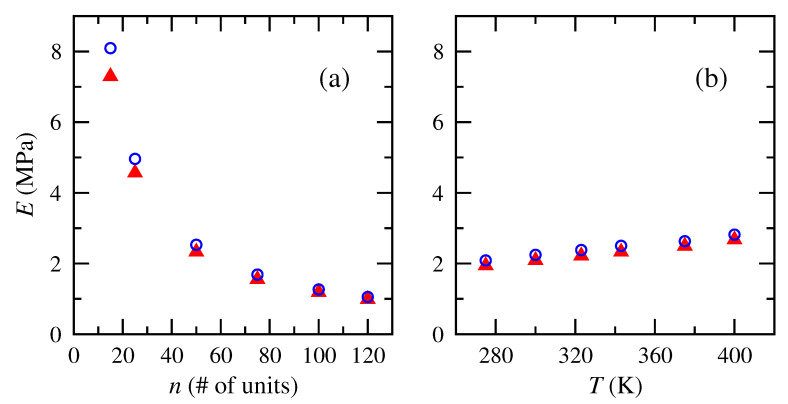
Tensile or Young’s moduli of cis- and trans-1,4-polybutadiene RIS chains of (**a**) different numbers of repeat units at 343 K and (**b**) different temperatures for 50 repeat units. Numerical model results: cis (▴ red); trans (∘ blue).

Computations of Young’s moduli from the slope of the linear regime of the stress–strain plots for cis- and trans-1,4-polybutadiene chains of different numbers of repeat units and temperatures are shown in Figure 8. Moduli significantly decreased with increasing number of repeat units and increased linearly with increasing temperature. Both arise from the scaling relationships that are incorporated into the normalized stress–strain plot. The chains with fewer repeat units correspond to subchains of lower molecular weight between cross-links. In a network polymer, this corresponds to a more tightly cross-linked chain network. Tonelli and Andrady [5,40] reported a similar rise in modulus with ever shorter chains between cross links in PDMS. Polybutadiene networks that incorporated increased extents of cross linking of the same starting material increased in modulus as a consequence of the shorter elastically active chains that arose [37].

The reported moduli are of the correct order-of-magnitude for elastomers. Textbooks on rubber elasticity [3,64] and polymer engineering [4] have reported Young’s modulus of polybutadiene to be ∼1 to 3 MPa, and the numerical results here fall within that range for all except the shortest chains. The 50-unit size reported here for multiple temperatures corresponds roughly to a molecular weight between entanglements.

To compare the RIS results more extensively with experimental results for polybutadiene, stress–deformation data were extracted from graphical depictions in the literature. Dossin and Graessley [37] presented Mooney–Rivlin plots for radiation cross-linked networks at 298.15 K that were made from two different polybutadiene starting materials. The ratio $\sigma_{\mathrm{engr}}/\left(\lambda -1/\lambda^{2}\right)$ shown in their results is equivalent to the ratio here of $\sigma_{\mathrm{true}}/\left(\lambda^{2}-1/\lambda \right)$ under an assumption that area shrinks as $A_{0}/\lambda$ during uniaxial deformation. Chen et al. [41] reported true stress vs. Hencky strain and deformation ratio at temperatures that included 273.15 and 298.15 K within their studies of stress-induced crystallization in a cross-linked polybutadiene rubber.

**Figure 9:**
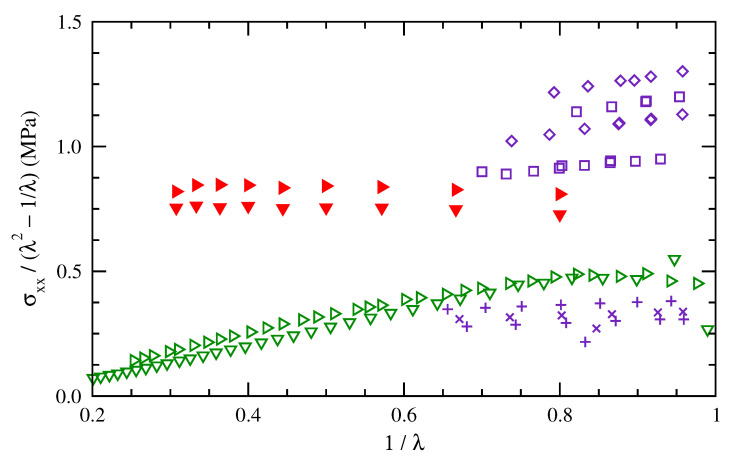
Mooney–Rivlin plot of normalized stress for polybutadiene as measured at 298.15 K for networks cross linked from two different starting materials (□, ⋄ and ×, + blue) [37] and at 273.15 and 298.15 K for a different cross-linked polybutadiene rubber (▹, ▿ green) [41]. Simulation results are for cis-1,4-polybutadiene of 50 repeat units at 275 and 300 K (▾, ▸ red).

Figure 9 compares the results here for cis-1,4-polybutadiene chains of 50 repeat units at 275 and 300 K with those results from the literature. The experimental data at 298 K show a range of normalized stresses (*y*-axis), indicating the effects of different cross link densities and effective molecular weights. The simulation results fall between data from the two different networks of Dossin and Graessley [37] and above the data of Chen et al. [41]. Both the simulation results and the experimental data of ref [41] show only a small rise in normalized stress with increased temperature. In terms of Mooney–Rivlin parameters, the simulation results demonstrate less of a dependence on 1/λ. Svaneborg et al. [42] found a similarly small dependence in their simulation results for shorter vs. longer chains. The tube model has been interpreted [24] as indicating that entanglements contribute to both the slope and intercept of a Mooney–Rivlin plot, while cross linking affects the intercept much more. Some differences between the simulation results and the experimental data are potentially a consequence of the absence of entanglements in the simulation method.

**Figure 10:**
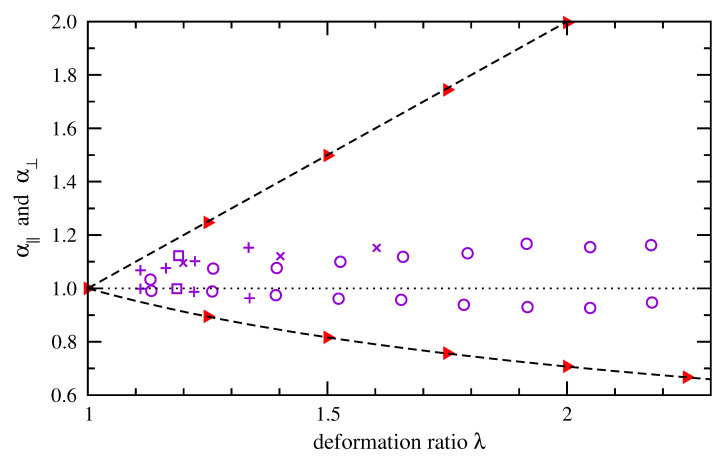
Ratios of root-mean-squared contributions to end-to-end distance in directions parallel and perpendicular to the deformation direction, normalized by contributions in the absence of deformation. Simulation results depict cis-1,4-polybutadiene of 50 repeat units at 300 K (▸ red). Equivalent radius of gyration data measured by neutron scattering [34] are shown for PDMS of increasing molecular weight (□; +; ×; ∘ violet). Dashed lines indicate changes expected for locally affine deformation. Parallel and perpendicular ratios are above and below the dotted line.

Experimental studies described in the introduction support a wide range of interpretations for how individual chains evolve in shape under macroscale deformation. As a test of one example, we show in Figure 10 how components of the mean-square end-to-end distance evolve under deformation in our calculations. We divide the end-to-end distance into parallel (*x*) and perpendicular (*y*, *z*) components relative to the deformation direction. Then, we compute averages over all chains at a given deformation ratio λ and compute ratios to the undeformed case (λ=1),

(33) $$\alpha_{\|}={\left(\frac{{\langle {r_{x}}^{2}\rangle }_{\mathrm{at}\;\lambda }}{{\langle {r_{x}}^{2}\rangle }_{\mathrm{at}\;\lambda =1}}\right)}^{1/2}\;\alpha_{⊥}={\left(\frac{{\langle {r_{y}}^{2}+{r_{z}}^{2}\rangle }_{\mathrm{at}\;\lambda }}{{\langle {r_{y}}^{2}+{r_{z}}^{2}\rangle }_{\mathrm{at}\;\lambda =1}}\right)}^{1/2}$$

Filled triangles (red) indicate our results for cis-1,4-polybutadiene of 50 repeat units at 300 K. Other data points indicate comparable ratios for radius of gyration of PDMS reported by Beltzung et al. [34] via neutron scattering measurements. The simulation results follow affine deformation precisely. The measurements show smaller shape changes for the PDMS chains, though the chains of lowest molecular weight (squares) are less far from exhibiting affine deformation. A similar deviation from affine deformation along an average polybutadiene chain during uniaxial deformation was reported by Naumova et al. [30] via NMR measurements. Alternate approaches for simulating changes in conformation probability density would be necessary to achieve closer agreement with these shape changes that have been measured experimentally.

## 4. Conclusions

The aim of this work was to develop a simulation method to predict mechanical properties of elastomers at various temperatures under deformation through explicit changes in chain conformation probability densities. We applied it to cis-1,4- and trans-1,4-polybutadiene chains of different numbers of repeat units. This approach expanded on prior work in which we generated distributions of end-to-end distance for individual chains. To create an initially isotropic orientation for the present work, all distances within this ensemble of chains were randomly distributed over the (+x,+y,+z) octant of a sphere. Then, all chains were uniaxially deformed affinely via extension along the *x*-direction and compression along the *y*- and *z*-directions. Probability density distributions of the chain end-to-end vectors changed due to this deformation, which resulted in changes in the elastic free energy of each ensemble of chains. The numerical method derived for computing the elastic free energy change of chain ensembles ultimately led to numerical evaluations of force and stress. For an ensemble of end-to-end distances that represented Gaussian chains, the results were in excellent agreement with analytical solutions. This demonstrated that the method achieved a successful numerical solution for a known case.

Next, the simulation method was applied to calculate mechanical properties of RIS chains. Ensembles of cis- and trans-1,4-polybutadiene chains that were generated under unperturbed conditions using Flory’s RIS method in previous work [58] were distributed in random directions and then were uniaxially extended. Elastic free energy changes, forces, and stresses were calculated using the newly developed method. Tension forces, compression forces, and tensile stresses increased with deformation. Normalized results emphasized scaling relationships for the forces and stresses as functions of molecular weight, chain density, and temperature. Differences in stresses and forces were minimal between cases that corresponded to cis- and trans-1,4-polybutadiene chains.

The slope of the stress–strain curves in the linear regime provided the Young’s modulus for each combination of chain type, number of repeat units, and temperature. Significant variations were observed in moduli as a function of the number of repeat units (i.e., molecular weight), while minor variations were observed with temperature. Chains with fewer repeat units demonstrated greater moduli, which was explained as corresponding to a much more tightly cross-linked network. Numerically computed moduli and stress–strain behavior as depicted on a Mooney–Rivlin plot were consistent with available experimental results. Averaged molecular-scale changes in conformation on a chain-by-chain basis differed from experimental results from the literature for PDMS chains, whose changes in shape were closer to the phantom model of network deformation.

## Data Availability

Data from this paper are available by contacting the corresponding author.
